# Roughening Plugger Points

**Published:** 1873-11

**Authors:** 


*An Easy Method of Roughening Plugger Points.*—Dr. W. S. Elliott, in the *Missouri Dental Journal*, describes a simple process which may prove useful to many practitioners. It is as follows:

"It has been suggested that a *broken* surface for a plugger point was in many respects superior to an ordinarily serrated one. This I believe to be true; but to break a point that it may be of an oval or spheroidal shape is no doubt impossible. A surface may, however, be produced which in all respects is equal to a broken one by the following method: First, file up the point to the shape desired and finish on a fine emery wheel; take now a piece of new emery paper of moderately coarse grade, lay it upon a well tempered anvil, and while the steel point is yet soft, place it upon the paper and with a smart blow of a hammer drive the point upon the emery: indentations will thus be made. Lift the point to a fresh surface on the paper and drive again; repeat the operation until the point is uniformly indented. For spherical points the paper may be bent at a right angle and placed in a similar angle of the anvil, when the *sides* of the point will receive the indentations equally as well defined as the extreme end. Harden without drawing the temper and examine under a glass."

---

